# Nurturing tomorrow’s leaders: The ISCB Student Council Symposia in 2018

**DOI:** 10.12688/f1000research.17739.1

**Published:** 2019-01-09

**Authors:** Daniele Parisi, Gabriel J. Olguín-Orellana, Eli J. Draizen, Nilson Da Rocha Coimbra, Nikolaos Papadopoulos, Susanne Kirchen, Yvonne Saara Gladbach, Numrah Fadra, Nazeefa Fatima, Aishwarya Alex Namasivayam, Sayane Shome, Dan DeBlasio, Alexander M. Monzon, Farzana Rahman, R. Gonzalo Parra

**Affiliations:** 1 ESAT-STADIUS, KU Leuven, Heverlee, Belgium; 2 Center for Bioinformatics and Molecular Simulations, Universidad de Talca, Talca, Chile; 3 Department of Biomedical Engineering, University of Virginia, Charlottesville, VA, USA; 4 Bioinformatics Graduate Program, Universidade Federal de Minas Gerais, Belo Horizonte, Brazil; 5 Quantitative and Computational Biology, Max Planck Institute for Biophysical Chemistry, Gottingen, Germany; 6 Luxembourg Centre for Systems Biomedicine (LCSB), University of Luxembourg, Esch-sur-Alzette, Luxembourg; 7 Institute for Biostatistics and Informatics in Medicine and Ageing Research, IBIMA - Rostock University Medical Center, Rostock, Germany; 8 Faculty of Biosciences, Heidelberg University, Heidelberg, Germany; 9 Division of Applied Bioinformatics, German Cancer Research Center and National Center for Tumor Diseases Heidelberg, Heidelberg, Germany; 10 Division of Biomedical Statistics and Informatics and Mayo Clinic and Department of Bioinformatics and Computational Biology, University of Minnesota, Minneapolis, MN, USA; 11 Department of Biology, Lund University, Lund, Sweden; 12 Bioinformatics and Computational Biology Program, Iowa State University, Ames, IA, USA; 13 Computational Biology Department, Carnegie Mellon University, Pittsburgh, PA, USA; 14 Department of Biomedical Sciences, University of Padova, Padova, Italy; 15 Genomics and Computational Biology Research Group, University of South Wales, Cardiff, UK; 16 Genome Biology Unit, European Molecular Biology Laboratory, Heidelberg, Germany

**Keywords:** symposia, ISCB, student council

## Abstract

The Student Council of the International Society for Computational Biology (ISCB-SC) is a student-focused organization for researchers from all early career levels of training (undergraduates, masters, PhDs and postdocs) that organizes bioinformatics and computational biology activities across the globe. Among its activities, the ISCB-SC organizes several symposia in different continents, many times, with the help of the Regional Student Groups (RSGs) that are based on each region. In this editorial we highlight various key moments and learned lessons from the 14th Student Council Symposium (SCS, Chicago, USA), the 5th European Student Council Symposium (ESCS, Athens, Greece) and the 3rd Latin American Student Council Symposium (LA-SCS, Viña del Mar, Chile).

## Introduction

The International Society for Computational Biology Student Council (ISCB-SC) is composed of and led by student and post-doctoral researchers. Since its origin, the ISCB-SC has been instrumental in organizing symposia, workshops, and seminars each year to introduce the newest computational biologists to the field and vice versa, as well as to empower them to communicate and participate in the community. The cornerstone of these events are the four annual symposia that accompany the major ISCB conferences (ISMB, ECCB, ISMB-LA, ISCB Africa). The symposia are headlined by keynote lectures from renowned researchers, who, apart from discussing science, usually also provide valuable career advices for the young audience. Organizing such meetings requires collective work that spans several months, where the organizing committee needs to accomplish multiple tasks in a timely fashion in order to meet the quality standards set by previous organizers. Being part of such a team requires motivation, commitment, resilience and the development of different leadership and soft skills.

In this article we highlight three such symposia that happened in 2018: the 14th Student Council Symposium (SCS) in Chicago, USA; the 5th European Student Council Symposium (ESCS) in Athens, Greece and the 3rd Latin American Student Council Symposium (LA-SCS) in Viña del Mar, Chile. In doing so, we also summarize the main goals of the symposia, our general structure, and how each of the individual events is tailored for its specific purpose.

### The networker scientist

The image of a scientist as a bespectacled genius with unruly hair, lab coat, clipboard in hand, isolated in a laboratory, surrounded by mysterious technology and fizzling test tubes seems ingrained in our culture ^1^. While this cliché may have held some truth in the past, a modern scientist needs much more than a sharp mind and good analytical skills in order to be successful in the current scientific system. Science, particularly at the interface of biological and computational disciplines, has become extremely complex and demands researchers from different fields to form interdisciplinary teams and to establish strategic and strong collaborations to reach sustainable, long-term success. Because of this, scientists need to develop a whole toolbox of both analytical and technical skills, but just as importantly, a set of soft skills that allow them to surf the complex waters of human interactions and leadership. Successful scientists are no longer fully enclosed in the laboratory performing experiments, but on the contrary spend a significant amount of their time networking, tweeting, blogging, writing divulgation books and so on. Keynote speakers at big conferences usually hold big personalities, they own the stage with their presence and captivate the audience with their communication skills. Regardless of how unreachable these scientific profiles might seem, all these people built their careers from scratch and had to work their way towards the position they occupy now. Additionally, in general, these researchers are hubs of large collaborative networks, and interacting with them benefits the student community by granting the opportunity to get in touch with closely and distantly related fields, expanding their minds to new career horizons ^2, 3^.

### Training a new generation of research leaders

Since 1997, the International Society for Computational Biology (ISCB) has organized meetings for gathering together the most world-renowned researchers in the fields of bioinformatics and computational biology. Early on, the ISCB directors realized that next to growing and strengthening the field, there lies a pressing need to nurture the next generation of computational biology researchers that would eventually take the lead from their hands. In 2005, the ISCB Student Council was created as a forum to bring together the next generation of computational biologists ^4^. The ISCB-SC is composed of Regional Student Groups (RSGs) located all around the world, several committees to handle the functioning of the council as a whole, and an executive team (ET) coordinating and directing their efforts. Every year, the ISCB-SC organizes various networking and training activities, among which there is a series of symposia that constitute the flagships of the organization ^5^. A chair and a co-chair lead each symposium’s organization and are in charge of building a team that executes different tasks that are required to generate a successful event. Organization of an ISCB-SC Symposium typically takes more than half a year of preparation. During this time the team is responsible for a diverse set of activities including contacting potential keynotes, fundraising, outreach communication, abstracts collection and evaluation, and social event planning. Throughout this process the chair and co-chair are responsible for keeping a fluent communication, keeping motivation and the collaborative mood among the team members, and dealing with unforeseen/problematic situations. Additionally, the chair, co-chair, and other key team members are responsible to lead the event and putting themselves in front of an audience for an entire day. For more than a decade, the ISCB-SC Symposia have been a platform for the new generation of computational biologists and has served as a source for the emergence of new leaders in our community.

### ISCB-SC Symposia in 2018

The ISCB-SC organizes four flagship events. The Student Council Symposium (SCS) is the only event held annually and has been organized since 2005 ^6– 8^. The European Student Council Symposium (ESCS) is held biennially and started in 2010 ^9^. Every two years SCS is held in Europe jointly with the ESCS as a jointly event ^5^. Since 2014 the Latin American Student Council Symposium (LA-SCS) has been biennial organized ^10, 11^ and since 2015 the African Student Council Symposium (SCS-Africa) has also taken place biennially ^12, 13^. In 2018 the ISCB-SC organized the 14th SCS in Chicago, USA, the 5th ESCS in Athens, Greece and the 3rd LA-SCS in Viña del Mar, Chile.

## Keynotes

Listening to a great keynote talk is always an inspiring experience. When attending a symposium, keynotes are one of the main attractions; it is a reason to pay the registration fee and spend an entire day in a given event. Summoning great keynote speakers is one of the most critical issues ISCB-SC symposia chairs have to face. Not only has the symposia series gathered top notch researchers in the field to deliver superb lectures, but it has also offered a unique environment where students can deeply interact with them. In a normal conference, with hundreds of senior scientists as part of the audience, asking a question or addressing a keynote personally after the talk is an intimidating experience for most young people. At the student council symposia, keynotes not only are accessible to the students during their talks and after, but also they prepare lectures that touch on topics beyond their scientific work including anecdotes about their career path that led them to become successful researchers in their fields. The 2018 Symposia were no exception.

In Chicago, SCS 2018 featured a talk by Dr. Lucia Peixoto from Washington State University titled “Learning-dependent chromatin remodeling highlights non-coding regulatory regions linked to Autism”. In addition to an excellent presentation, one of the great contributions of Dr. Peixoto is that she served as a co-founder of our very own Student Council back in 2004; she and her colleagues from the first ISCB-SC executive team paved the way for developing this organization and hence having her as a keynote after all these years is a big inspiration for all of us. The second SCS keynote lecture was delivered by Dr. Philip Bourne and named “Eight (so far) things I wish I had thought 40 years ago.” Dr. Bourne has been a key figure in bioinformatics, from being a professor at the UC San Diego to becoming the director of the University of Virginia’s Data Science Institute. In the style of his “Ten Simple Rules” pieces, he shared his views on how to become a successful biological data scientist, which he learned over the years. He also emphasized that science is a team sport, and that collaboration, management, communication and administration are just as important as the science. Phil Bourne is a great example of ‘the networker scientist.’

In Athens, the ESCS 2018 began with a talk by Dr. Anna Zhukova (Institut Pasteur, Paris), who presented her work on “modelling the spread of HIV-1 resistance mutations” as well as her career path. The second keynote was delivered by Dr. Julio Saez-Rodriguez from the University of Heidelberg. His research combines mathematical models with biological data to explain disease mechanisms, such as logical models of signaling networks trained on data from mass spectrometry and antibody assays. Dr. Saez-Rodriguez also highlighted the importance of collaboration, both on a personal basis and on a community level: his group collaborates with many experimental groups and regularly participates in DREAM challenges, a community effort that promotes large-scale cooperation to answer specific biological questions. When asked for career advice, he commented that his career was not the result of meticulous planning, but rather the drive to research important questions, academic excellence, and actively looking for opportunities.

In Viña del Mar, for the 3rd LA-SCS, the oral session began with Dr. Wendy Gonzalez, head of the Center for Bioinformatics and Molecular Simulations at Universidad de Talca. In her talk “Molecules Son: the dance I am trying to learn,” she reflected on the history of her experience in studying ion channel associated diseases. The title refers to Son, the genre of music and dance of her home country, Cuba; e.g. the drugs “dance” as they move into channels associated with neoplasm and atrial fibrillation. Later, Dr. Francisco Melo from the Faculty of Biological Sciences at Pontificia Universidad Católica de Chile, referred to his vision about past, present and future challenges in bioinformatics research from the perspective of being located in Latin America, through his talk titled: “A perspective about doing research in molecular Bioinformatics from Chile: from past to present (and future …) challenges”. Finally, attendees at LA-SCS boarded a rocket to explore the Astrobiology field with Dr. David S. Holmes, founder of the Iberoamerican Society for Bioinformatics (SOI-BIO) and Head of the Center for Bioinformatics and Genome Biology at Fundación Ciencia y Vida. During his talk “Earth environments as analogs for extraterrestrial life”, he explored examples of sites that provide information on how physical and chemical conditions interact to form environments conducive to life and how metagenomics is being used to tease out essential genes and metabolisms needed to tailor-make microbes for applications in extra-terrestrial environments.

## Student highlights

Student talks represent the core of the ISCB-SC symposia. Within this assigned time, young researchers in bioinformatics have the chance to practice their presentation skills and receive useful comments from other peers. The ISCB-SC symposia play an important role to prepare students for more prestigious, but also demanding, stages. This year the three ISCB-SC symposia contained extremely high caliber scientific talks from students coming from many different universities and institutes, thanks to the now well-known events that preceded these as well as the careful selection process.

SCS in Chicago featured 10 student talks and 34 poster presentations that were selected through a competitive review process. During the symposium, the presenters were judged by delegates using an anonymous voting scheme. Best presentation awards went to Carolin Loos (Helmholtz Zentrum München) and Ben Siranosian (Broad Institute); best poster awards went to Susanne Pieschner (Helmholtz Zentrum München), Michael Scherer (Max Planck Institute for Informatics), and Susanne Kirchen (University of Luxembourg). We consider useful to give our audience of students and researchers the opportunity to critically evaluate and take a main role in the review process.

For ESCS 2018, 11 projects were presented as full talks and six in a 5-minute flash talk format. The decision for awarding the best talk and the best poster was made by all 50 participants, again by anonymous vote. The best talk award was given to Melissa Adasme (BIOTEC TU Dresden), for her talk “From malaria to cancer, computational drug repositioning of amodiaquine using PLIP interaction patterns”. The two best poster prizes went to Neetika Nath (University Medicine Greifswald) and Dilip Ariyur Durai (Max Planck Institute for Informatics).

During LA-SCS, the Student Council awarded the three most outstanding presentations. The jury was confirmed by the chairs of the symposium, who selected the winners among the 9 student talks and 16 poster presentations. The best student talk award was conferred to Juanita Gil (Universidad de los Andes), with her talk titled “Accurate, efficient and user-friendly simulation and mutation calling for TILLING experiments”. The best poster award was received by Mauricio Bedoya (Universidad de Talca) for his work “Relevance of extracellular portals in the potassium K2P ion channel conduction mechanism”. An honor mention was given to David Medina (Universidad de Chile) for his talk “VHL-Hunter, a web service for classification of clinical relevance in single point mutations in Von Hippel-Lindau disease”.

## A novelty in the student symposium structure: round table “Bioethics and bioinformatics”

For the first time in ISCB-SC symposia history, ESCS 2018 hosted a roundtable about bioethics and its importance in the field of bioinformatics. The roundtable aimed to increase students’ awareness about current popular topics such as data sharing, data protection and social hazards of technology. To start, Dr. Yves Moreau delivered a talk titled “‘Build it and they will come’, or how will we prevent abuses of clinical genomic databases?” followed by Dr. Mahsa Shabani who talked about “Ethical concerns associated with data sharing in biomedical sciences”. After, Dr. Moreau and Dr. Shabani were joined by the two ESCS keynotes, Dr. Saez Rodriguez and Dr. Zhukova, for an open discussion about bioethical issues in bioinformatics and computational biology in general. The students engaged in a stimulating and vivid discussion about the impact of bioinformatics in future society. We encourage future chairs and co-chairs to organize similar events that stimulate critical thinking about the next big trends in our field.

## Challenges and lessons learned

Success is the result of planning, hard work, determination, foresight, and a little bit of luck. However, regardless of how much planning or how much experience the organizers or their advisors have, unforeseen situations will often arise and challenge the team’s resilience and versatility. Although these conflicting situations can be discouraging and time consuming they present valuable learning opportunities that serve to strengthen the operational skills of the members of the team. The ISCB-SC has previously discussed the important role of challenging situations in the road to success and this still holds true: *“difference between those who succeed and those who abandon their projects lies in their response to adversity”* ^14^.

It is to be expected that problems will arise, possibly from the very beginning of the conference organization and, therefore, the team needs to come up with feasible and timely solutions. For many of the chairs and co-chairs, 2018 was their first time organizing a symposium. Here we share different stories about challenging situations and how we overcame them. We aim to encourage next chairs and co-chairs are prepared for when things do not go according to the initial plan. Many of these situations apply to all symposia while others are symposium specific.

### Risk assessment and mitigation

The organization of a symposium is, on its whole, an exercise in risk assessment and mitigation. Things deviate from the plan all the time; there are scheduling conflicts between team meetings and unexpected academic obligations, a team member cannot meet a deadline, delegates have forgotten their posters, a student speaker or keynote cannot attend due to health or visa issues, a keynote speaker goes missing last-minute and many other examples. The early setbacks teach you to take complications like those into account. By the end of the project you learned to have a contingency plan and you have extensively practiced on improvising for all those problems you could not foresee.

### Importance of interpersonal cooperation

On the other hand, these setbacks foster team spirit and help you to improve as a team player. It is impossible for the symposium chairs to personally take care of everything - after all they are volunteers as well and have their own academic obligations. You learn to encourage and delegate responsibility. You cultivate flexibility by jumping in to cover for other team members when needed, and they do it in turn.

Team cooperation can only be achieved by mutual respect and focus on the common goal. Keeping this in mind will help you navigate differences in opinion and learn from them, effectively managing conflicts within the team and using them constructively instead of letting them derail the effort. This often requires the flexibility to accept solutions and initiatives that don’t agree with your own vision, as long as they benefit the team. It is important to establish objective criteria for success and make evaluations according to them, and not according to how you would have done things personally.

When coordinating a team with many members, time zones and obligations, fluent and organized communication is crucial. It is mandatory to have a centralized communication channel where messages can be easily delivered to all team members and information is quickly available to everyone at any time. This makes it easier to coordinate and keep track of progress, and also provides records of discussions and meetings, a valuable and easily accessible resource for future organizers.

### Importance of promotion and outreach

One of the most crucial challenges is related to how to promote the event and reach a wide audience. At the end of the day, regardless of how good the keynotes, the venue and the organization are, much of the success is reflected on the amount of attendants that register and present their work at the event. The Student Council might not be on everyone’s radar, or people might not be aware of how the symposium is different from the main conference. While we mainly used social media (Twitter and Facebook), there is quite room for improvement. Most RSGs utilize social media and, therefore, could work in a more coordinated manner spreading the important information of the symposia. This requires an active role of the outreach committee, calling to the RSG community managers to share the announcements. Even though RSGs can cover large geographic regions, everyone needs to work together in the next years to find ways to promote participation of countries not present until now.

### Finding the keynotes

This is perhaps the most important task the team needs to address. Over the years, we learned that if you aim to have the reputed scientists in the field as keynote speakers, you need to contact them as early as possible—it helps to network ahead of time so they know who you are before inviting. An early list of keynote speaker candidates is essential. Subsequently, just try to contact them one by one until the desired number of keynotes is confirmed. It is also important to have a backup plan in case things don’t work out, such as week-of cancellations. While selecting keynotes, the ISCB-SC does its best to maintain gender balance and diversity, i.e. inviting women, people from different ethnicities, and members from other underrepresented communities. In the US, the field of bioinformatics is unfortunately dominated by white men. In order to change this, we believe that we need to highlight diversity and show newcomers into the field that everyone is accepted and welcome ^15^.

### Finding sponsors

Sometimes finding sponsors can be challenging. Some sponsors may not have heard of the Student Council and/or the parent organization ISCB. We suggest that the search for sponsors receives equal importance to keynote search and that the organizing committee also considers sponsors from outside the country where the symposium takes place. It is important to know that different companies of institutions have specific times in the year for when they need to decide on which sponsoring activities they will invest their money. Because of this an organized schedule for contacting sponsors as well as following up with them is needed. This year was a real challenge since we had to find sponsors for three different symposia. It is important to keep good relations with the sponsors and maintain collaborations with them after the event is over.

### Unforeseen hurdles

Many unforeseen problems can and will occur during the symposium. Therefore, it is very important to be prepared: from lack of sponsors to student and speaker last minute changes/cancellations. Even satellite activities can give you a huge headache: e.g. when you realize you booked the social event for the wrong time or the catering forgets to bring the required food and drinks.

Problematic situations can arise from the most diverse internal or external sources. This year important economic processes happened in the Latin American continent, with many currencies being devalued in respect to the American dollar. This severely affected the organization and success of LA-SCS due to the registration cost. Since Chile is considered a High Income Country, based on World Bank ranking of economies ^16^, the high symposium registration price was hard to pay for both national and international students from the region and had large consequences on the composition of attendants to the event. This forced the organizers to reduce the registration fees to a much lower level compared to standard costs on this type of symposium and to dedicate a substantial proportion of the budget to provide travel fellowships.

## Conclusion

The importance of transferable skills such as teamwork and management, networking, leadership and effective communication, has been prioritized to achieve success both in the academic and scientific community ^17^, with many researches showing a correlation between their networking and career outcomes ^18^. In sight of this, it is crucial for young researchers to engage themselves in organizing and attending student symposia, where the development of transferable skills is proactively encouraged ^19^, and the community increases its cohesiveness ^20^. These events represent important opportunities for students to present their own work to a community of peers, and gain skills that are indispensable on peer-review processes.

Co-organizing a symposium positively impacts on every member’s networking opportunities. All team members are highly motivated, high achieving PhD students and postdocs who will become future colleagues and collaborators, future principal investigators or future board members. Symposia organizers get the chance to interact with high-profile scientists who are invited to give keynote talks. Cultivating relationships with sponsors increases your network and fosters collaboration at the same time that introduces the team members on how to deal with the fundamental task of fundraising for organizing scientific events. Certainly, the Student Council Symposia represent the major networking and education activities oriented to bioinformatics students around the world. Despite the geographical distance among the venues where the different events have been organized so far and the different cultures represented by the attendants, from its first edition they had managed its purpose: promote the develop good relationships between students, keynote speakers, sponsors, universities and scientific institutes.

Since the main ISCB conferences are large, it is important to help PhD students find peers early on so the main conference doesn’t feel overwhelming. We strive to make it an accepting and welcoming community; ISCB-SC symposia are opportunities to practice giving talks in front of a large audience while fostering positive criticism and the establishment of new collaborations. This is further enhanced during the coffee breaks, the social event and a relaxed and stress-free atmosphere.

Time steadily advances and generational change is inexorable. In the next decades, new researchers will have to take over the command of our scientific community with the huge responsibility of keeping up to the level current board members have achieved. Conditions for international scientific development can become quite complex in a world where financial resources need to be carefully assigned, migration rules can change from one day to the other, gender policies are adapting to modern times where inclusion is a must and minorities need to be heard and respected. Moreover, individuals need to develop their skills in a much broader way to survive in a scientific system that becomes more competitive each day. Tomorrow’s leaders need to become aware of all these topics and have to be able to correctly manage not only scientific related problems but also social related ones. These leading people will often need to decide on subjects that are of political nature that need to be addressed with precision in order to defend the community’s interests. So far, leaders cannot be collected from trees in a field or grown up in a petri dish, so we need to nurture them in the old-fashioned way by training, teaching and learning from each other. The ISCB-SC symposia have gathered the young generation of computational biology for more than a decade and have helped to produce researchers that are slowly getting into spotlight positions in the community. Many of tomorrow’s leaders will necessarily emerge from the ISCB-SC and one of those can be you. Get involved and help us shape the future of this amazing community.

## Future editions

The ISCB-SC Symposia series have become a successful platform to collaborate, train and nurture the future bioinformaticians who will pave the way to strengthen bioinformatics and computational biology community. Following the success of previous symposium, the future editions of symposia are planned for SCS2019 in Basel, Switzerland, SCS2020 in Toronto, Canada, ESCS 2020 in Sitgens, Spain, plus editions of SCS-Africa in 2019 and LA-SCS in 2020.

## Data availability

No data is associated with this article.
